# Overexpression of HMGB3 and its prognostic value in breast cancer

**DOI:** 10.3389/fonc.2022.1048921

**Published:** 2022-12-22

**Authors:** Xiaomei Zhou, Qu Zhang, Gai Liang, Xinjun Liang, Bo Luo

**Affiliations:** ^1^ Department of Radiotherapy Center, Hubei Cancer Hospital, The Seventh Clinical School Affiliated of Tongji Medical College, Huazhong University of Science and Technology, Wuhan, China; ^2^ Department of Abdominal Oncology, Hubei Cancer Hospital, The Seventh Clinical School Affiliated of Tongji Medical College, Huazhong University of Science and Technology, Wuhan, China

**Keywords:** breast cancer, HMGB3, prognosis, biomarker, bioinformatic analysis

## Abstract

**Background:**

High mobility group protein B3 (HMGB3) is abundantly expressed in a number of malignancies, contributing to tumor cell growth and predicting poor outcomes. More research on the connection between HMGB3 and breast cancer is needed. The prognostic significance of HMGB3 in breast cancer was examined and validated in this study.

**Methods:**

Using The Cancer Genome Atlas (TCGA) database RNA sequencing and clinical data, we investigated the associations between HMGB3 expression and tumor mutations, prognosis, and immune infiltration in breast cancer. The Gene Expression Profiling Interactive Analysis (GEPIA), Tumor Immune Estimation Resource (TIMER), breast cancer gene-expression miner (bc-GenExMiner), UALCAN, OncoLnc, cBio Cancer Genomics Portal (cBioPortal), and LinkedOmics databases were applied to examine the levels of expression, mutation, coexpression, and immune correlation of HMGB3 in breast cancer. cBioPortal and the Database for Annotation, Visualization, and Integrated Discovery (DAVID) were used for coexpression and enrichment analyses, respectively. Experimental tests and a separate cohort of breast cancer patients in our center were used for validation. To determine independent risk factors affecting breast carcinoma prognosis, multivariate Cox regression analysis was performed. The Kaplan-Meier method was applied to analyze the connection between HMGB3 expression and overall survival time in breast cancer.

**Results:**

Pan-cancer investigation using the GEPIA and UALCAN databases revealed a high level of HMGB3 expression in different malignancies, including breast cancer. HMGB3 might be a potential diagnostic biomarker, according to the receiver operating characteristic (ROC) curve (AUC=0.932). And immunohistochemistry confirmed higher HMGB3 protein expression in breast cancer tissues in clinical samples. Experimental tests also showed that breast cancer cells have higher expression of HMGB3, and knockdown of HMGB3 can promote the proliferation of breast cancer cells and increase sensitivity to chemotherapy. Human epidermal growth factor receptor 2 (HER2), Nottingham Prognostic Index (NPI), basal-like status, nodal status (N+), triple-negative status, and Scarff-Bloom-Richardson (SBR) grade all showed positive correlations with HMGB3 expression. Conversely, HMGB3 expression was negatively associated with the expression of estrogen receptor (ER) and progesterone receptor (PR) in breast cancer. Breast cancer patients with high HMGB3 expression had poor overall survival, which was validated by an analysis of a separate cohort of breast cancer patients in our center. Cox regression analysis identified high HMGB3 expression as an independently associated risk factor for breast carcinoma. The amount of immunological infiltration was substantially linked with the high expression of HMGB3. The chromosome centromeric region, ATPase activity, and the cell cycle are critical areas where HMGB3 is involved, according to enrichment analysis. Therefore, we suspected that HMGB3 might be a potential biomarker for detecting and treating breast carcinoma.

**Conclusion:**

Breast cancer tissues had higher HMGB3 expression than normal breast tissues. HMGB3 overexpression may serve as an indicator for poor breast cancer outcomes.

## Introduction

The most prevalent disease in women is breast cancer (BRCA), with an estimated approximately 2.3 million new cases identified in 2020 (1). The molecular subtypes of BRCA can be identified based on the levels of mRNA gene expression (Luminal A, Luminal B, basal-like, and HER2-enriched) (2). Some BRCA patients, especially those with the basal-like subtype, tend to develop metastases and have a poor prognosis despite major improvements in surgery, endocrine therapy, radiotherapy, chemotherapy, and targeted therapy (3). Although many studies have explored the progression of BRCA, the detailed mechanism has not been fully elucidated. Consequently, finding new biomarkers for the prognosis of BRCA is crucial for early detection and successful treatment. Chromatin-binding protein HMGB3 belongs to the X cluster (Xq28) family. It is found in the nucleus, cytoplasm, and chromosomes and is mostly expressed in bone marrow hematopoietic stem cells and embryonic cells, while it is absent or barely expressed in other normal tissues (4). Many studies have discovered a clear connection between the aberrant expression of HMGB3 and the incidence of various tumors, and HMGB3 is abundantly expressed in a variety of malignancies (including lung cancer, gastric cancer, bladder urothelial carcinoma, esophageal carcinoma and prostate adenocarcinoma) (5–8). Esophageal squamous cell carcinoma and bladder cancer patients with high HMGB3 expression have poor prognoses (6, 9). Some researchers also showed that HMGB3 overexpression is possibly associated with BRCA; however, it is unclear whether its overexpression has an impact on the prognosis of BRCA (10). Furthermore, it is currently unclear whether HMGB3 can be utilized as a predictive biomarker for BRCA. Evaluating the predictive value of HMGB3 expression in breast cancer is the goal of this investigation.

## Methods

### Data processing

The Cancer Genome Atlas (TCGA, http://cancergenome.nih.gov/) database was used to download the breast cancer mRNA expression data and clinical information. We collated the raw data and divided breast cancer patients into high and low expression groups based on median HMGB3 expression. It was further compared with clinicopathological characteristics and prognostic data of breast cancer patients.

### Analysis of gene and protein expression

Tumor Immune Estimation Resource (TIMER) is an integrated tool for the systematic assessment of immune infiltration in a wide range of cancer types. It includes information on somatic mutations, gene expression, clinical outcomes, and tumor-immune system characterization. A total of 10,897 samples representing 32 distinct cancer types are available in The Cancer Genome Atlas (TCGA) database to calculate the degree of immune infiltration. For the 32 types of cancer obtained from the TCGA project, we entered HMGB3 into the “Gene-DE” module of the TIMER2.0 site and examined how the expression of HMGB3 varied between tumor and nearby normal tissues (11).

A web-based program called Gene Expression Profiling Interactive Analysis (GEPIA) can perform quick and adaptable tasks such as differential expression analysis, patient survival analysis, correlation analysis, and related gene recognition (12). We examined HMGB3 expression in TCGA tumors, using matching normal and Genotype-Tissue Expression (GETx) data from TCGA as controls.

By integrating clinical information from 31 different types of cancer with TCGA RNA-seq date, UALCAN provides an interactive web-based tool to analyze TCGA gene expression data in depth (13). Here, utilizing information from the Clinical Proteomic Tumor Analysis Consortium (CPTAC) (14), we examined the comparative expression of HMGB3 in normal and tumor samples.

Large amounts of breast cancer genomic information are available in breast cancer gene- expression miner (bc-GenExMiner) v4.8, which is capable of statistical analyses of expression, correlation, and prognosis. Using bc-GenExMiner v4.8, the associations of the HMGB3 gene and the clinicopathological features of breast cancer were examined (15–17).

### Immunohistochemistry (IHC)

Retrospective collection and analysis of paraffin-embedded breast cancer samples from patients at Hubei Cancer Hospital was performed for this study. According to the ethics committee’s criteria, each patient gave their informed consent (LLHBCH2020LW-022). We collected tissue samples of breast malignancy as well as normal tissues adjacent to cancer from 210 patients who were pathologically confirmed to have malignant tumors with metastases in our center. The paraffin sections were dewaxed and hydrated with xylene. Antigen repair was performed using EDTA antigen repair solution (pH=9.0), and sample blocking was carried out using regular bovine serum blocking solution at room temperature for 30 min. At 4°C for an overnight incubation, the sections were treated with the primary antibody (anti-HMGB3 rabbit monoclonal antibody diluted 1:300). The goat anti-rabbit secondary antibody that had been coated with horseradish peroxidase was incubated for 50 min at room temperature. Under a microscope, the 3,3’-diaminobenzidine (DAB) staining intensity was evaluated. Neutral glue was used to encapsulate hematoxylin-stained cells, and the cells were then examined under a microscope. The expression of HMGB3 was comprehensively scored according to the percentage of positive cells and the staining intensity of stained tumor cells. The staining intensity was scored as “0” (no staining), “1” (weakly stained), “2” (moderately stained) or “3” (strongly stained). The score for determining the percentage of positive cells was as follows: 0-5% positive cells, 0; 5-25% positive cells, 1; 25-50% positive cells, 2;50-75% positive cells, 3; and 75-100% positive cells, 4. The staining evaluation of HMGB3 was as follows: a final staining score of <3 was “-”; a final staining score of 3 was “+”; a final staining score of 4 was “++”; and a final staining score of ≥5 was “+++”. All patients were divided into two groups of low and high HMGB3 expression according to an IHC score of 3.

### Cytotoxicity testing

Breast cells in the early log phase were trypsinized and plated in 96-well plates at a density of 4000 cells/well. After 24 h, the medium was removed and replaced with fresh medium containing different concentrations of drugs. Cell viability was measured for 24 h by using a methylthiazolyldiphenyl-tetrazolium bromide (MTT) kit (Sigma-Aldrich) following the manufacturer’s instructions. In brief, 10 µl of MTT was added to each well at a final concentration of 0.5 mg/ml. Then, the cells were incubated for 4 h at 37°C. Subsequently, the media/MTT mixture was removed, and 150 µl of dimethyl sulfoxide (DMSO) was added to dissolve the MTT crystals (formazan). The absorbance of the sample at 490 nm was read using a Bio-Rad microplate reader (model 630; Hercules, CA, USA).

### siRNA and cell transfection

Cells were transfected using a liposome delivery system (Cat. # 11668027, Thermo Fisher Scientific). For the transfection of small interfering RNA (siRNA), siRNA oligonucleotides targeting HMGB3 and nonspecific siRNA oligonucleotides were purchased from GenePharma (China): 5′-GGUCUUCGCCUUGAUUCAUTT-3′ and 5′-AUGAUAUAAGGCGAAGACCTT-3′. The siRNA oligonucleotides were transfected into BRCA cells using liposomes according to the standard protocol provided by the manufacturer. Forty-eight hours after transfection, cells were harvested and analyzed by western blotting or used in further experiments.

### Survival prognosis analysis

OncoLnc, an online repository of TCGA survival information connected to mRNA expression levels, was used to study the prognostic significance of these data (18), and evaluate the connection between HMGB3 and BRCA patient survival. The cutoff value was set at 50% to interpret HMGB3 expression data correctly. Using the GEPIA2.0 database and Kaplan-Meier Plotter (19), we further confirmed the predictive relevance of HMGB3 expression in BRCA tissue. We chose a custom cancer type, entered the HMGB3 gene into the “survival analysis” module, and analyzed overall survival (OS) using log-rank tests.

### The relationship between HMGB3 and immune cell infiltrates

Several immune cells that infiltrate tumors were predicted using the TIMER database on breast cancer tissue. The number of invading immune cells, including CD4+ T cells, CD8+ T cells, B cells, neutrophils, dendritic cells, and macrophages as well as the level of HMGB3 gene expression were measured. Through the use of the correction module in the TIMER database, we investigated the expression of HMGB3 and genetic signatures of immune cell subpopulations. Next, we estimated the statistical significance and computed the Spearman’s correlation coefficient.

### Analyses of coexpressed genes and genetic alterations

The mutation module of cBioPortal was applied to visualize HMGB3 mutations in BRCA (20). To identify the genes that were coexpressed with HMGB3, Pearson correlation coefficient analysis was performed on multiomics data from 32 distinct cancer types using the LinkedOmics database (21).

### HMGB3 related gene enrichment analysis

Fifty HMGB3-related genes were found in GEPIA. Database for Annotation, Visualization, and Integrated Discovery (DAVID) was used to carry out enrichment analyses based on the 50 related genes using the Gene Ontology (GO) and Kyoto Encyclopedia of Gene and Genomes (KEGG) databases (22). The enrichment results were then visualized for bioinformatics analyses using the Gene Ontology chord plot tool.

### Statistical analysis

Statistical analysis was performed using SPSS (IBM, USA) software. The survival times of the HMGB3 high and low expression groups were compared using Kaplan-Meier analysis, and the p value was calculated using the log-rank test. By using the GEPIA database and OncoLnc web tool, survival curves were produced. Using univariate Cox regression analysis, the clinicopathological features of breast cancer and the connection between HMGB3 and OS time were investigated. To identify independent risk factors affecting breast cancer patient prognosis, multivariate Cox regression analysis was employed to examine the factors influencing breast cancer patient survival in univariate Cox regression analysis. We defined statistical significance as p<0.05.

## Results

### Expression of HMGB3 in BRCA and experimental validation

**Figure 1:**
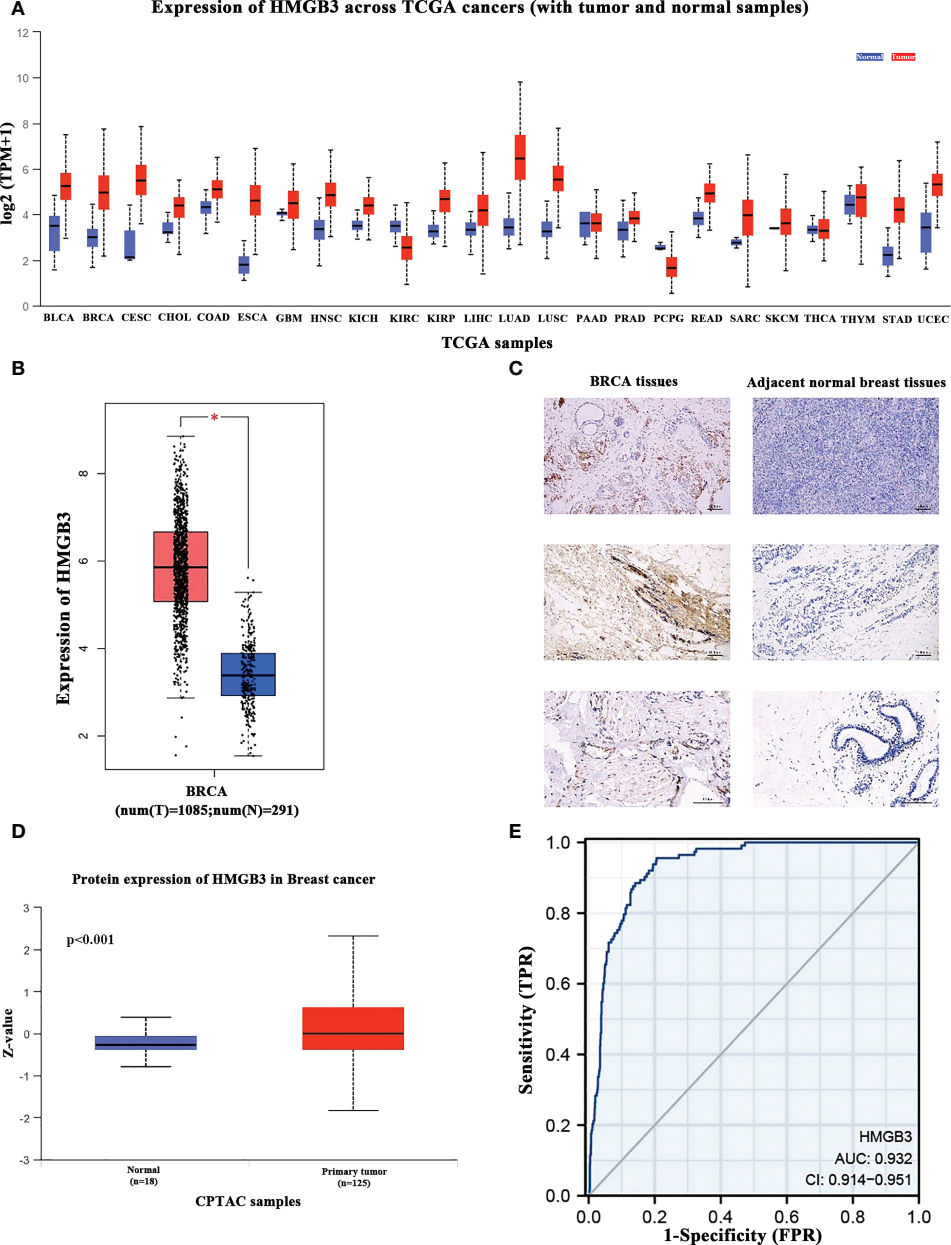
HMGB3 expression levels in various cancer types. **(A)** UALCAN database was used to analyze the levels of HMGB3 expression in various types of cancer tissues and their matched normal tissues. **(B)** Breast cancer tissues have higher levels of HMGB3 than normal tissues, according to Gene Expression Profiling Interaction Analysis (GEPIA); *p<0.05. **(C)** HMGB3 immunohistochemical staining of BRCA tissues and adjacent normal breast tissues. Brown indicates the intensity of the expressed protein. **(D)** The presence of the HMGB3 protein in breast cancer. **(E)** Receiver operating characteristic curve of HMGB3 in BRCA. False-positive rates are represented on the X-axis, and true-positive rates are represented on the Y-axis.

**Figure 2:**
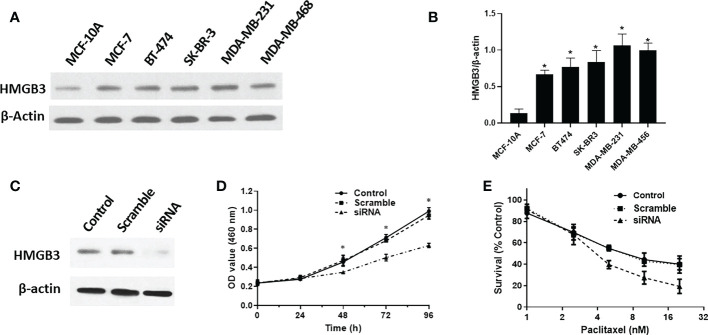
HMGB3 expression is correlated with proliferation and drug resistance. **(A, B)** Expression of HMGB3 in the human breast epithelial cell line MCF-10A and BC cell line through western blot analysis. **(C, D)** Cell proliferation was significantly suppressed in SK-BR-3 breast cancer cells transfected with siRNA. **(E)** Drug resistance to paclitaxel was reduced after siRNA mediated inhibition of HMGB3 expression. The results are presented as the mean ± SD. *P < 0.05.

We analyzed the levels of HMGB3 mRNA expression in all TCGA tumors using the UALCAN database to identify the variation in HMGB3 expression among tumor and nearby healthy tissues. The results revealed that most tumors overexpressed HMGB3, including breast cancer (BRCA), pheochromocytoma and paraganglioma (PCPG), cervical squamous cell carcinoma and endocervical adenocarcinoma (CESC), bladder urothelial carcinoma (BLCA), cholangiocarcinoma (CHOL), colon adenocarcinoma (COAD), head and neck squamous cell carcinoma (HNSC), kidney chromophobe (KICH), kidney renal papillary cell carcinoma (KIRP), kidney renal clear cell carcinoma (KIRC), esophageal carcinoma (ESCA), lung adenocarcinoma (LUAD), liver hepatocellular carcinoma (LIHC), lung squamous cell carcinoma (LUSC), rectum adenocarcinoma (READ), prostate adenocarcinoma (PRAD), uterine corpus endometrial carcinoma (UCEC), and stomach adenocarcinoma (STAD) (**Figure 1A**). Moreover, we used normal tissues from the GTEx dataset as controls to compare HMGB3 expression in tumor and normal BRCA tissues. Breast cancers showed elevated levels of HMGB3 expression, as shown in **Figure 1B**. The CPTAC also showed that breast cancer tissues had higher levels of HMGB3 protein expression (**Figure 1D**). A ROC curve was also employed to assess the viability of discriminating the HMGB3 expression levels between normal and malignant breast tissues. The test quality was represented by an area under the ROC curve (AUC) of 0.932 (**Figure 1E**). Additionally, we used IHC to confirm higher HMGB3 expression in breast cancer tissues than in adjacent normal breast tissues. Compared to normal tissues, BRCA tissues showed higher expression of HMGB3 (**Figure 1C**). Various breast cancer cell lines were also used to validate the expression differences between breast epithelial cells and BRCA cells (**Figures 2A, B**). And *in vitro* study showed that the growth of BRCA cells was inhibited and drug sensitivity to paclitaxel was increased after knockdown of HMGB3 expression (**Figures 2C–E**).

### Expression of HMGB3 and clinicopathological parameters

**Figure 3:**
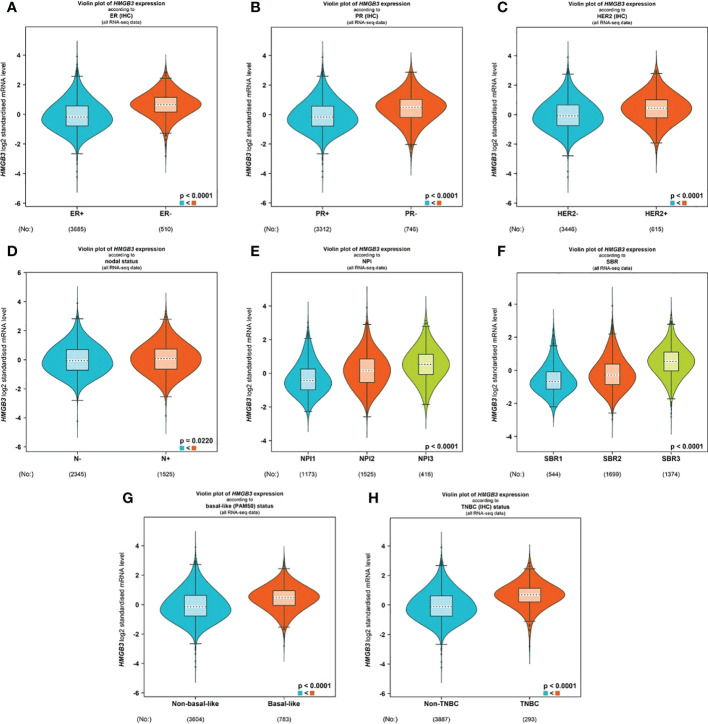
Correlation between HMGB3 expression and clinicopathological parameters. Using the software bc-GenExMiner v4.8, a violin plot was created to show the correlations between the expression of HMGB3 and several clinical and pathological markers. Information is displayed for the following variables: ER **(A)**, PR **(B)**, HER2 **(C)**, nodal status **(D)**, NPI **(E)**, SBR **(F)**, basal-like status **(G)**, and TNBC status **(H)**. ER, estrogen receptor; PR, progesterone receptor; HER2, human epidermal growth factor receptor 2; NPI, Nottingham Prognostic Index; SBR, Scarff-Bloom-Richardson; TNBC, triple-negative breast cancer.

With the use of the bc-GenExMiner v4.8 database, we examined the connection between HMGB3 and clinical and pathological signatures of breast cancer. ER and PR (p<0.0001) were shown to be adversely linked with HMGB3 expression (**Figures 3A, B**). However, the expression of HMGB3 was higher in the HER2 positive group than in the HER2 negative group(p<0.0001) (**Figure 3C**). Higher levels of HMGB3 were found in breast cancer patients with positive nodal status (N+) than in those with negative nodal status (N-) (p=0.0220) (**Figure 3D**). Increased Nottingham Prognostic Index (NPI) values were linked to higher expression of HMGB3 in the study (p<0.0001) (**Figure 3E**). A histological grade known as the Scarff-Bloom-Richardson (SBR) grade is used to assess the mitotic index, nuclear pleiomorphism characteristics, and tubule development. Patients with BRCA who had a higher SBR grade tended to have higher HMGB3 expression (p<0.0001) (**Figure 3F**). Additionally, we discovered that HMGB3 expression levels were substantially greater in the basal-like subtype than in the nonbasal-like subtype (p<0.0001), and individuals with triple-negative breast cancer (TNBC) also showed the same pattern of change (p<0.0001) (**Figures 3G, H**).

We also analyzed the connection between HMGB3 expression levels and clinicopathologic features in BRCA patients using logistic regression. According to the results of the study in **Table 1**, the following variables were significantly correlated with the expression levels of HMGB3: ER status (OR=0.207 for positive vs. negative), HER2 status (OR=1.550 for positive vs. negative), PR status (OR=0.393 for positive vs. negative), T classification (OR=2.328 for T4 vs. T1) and N classification (OR=1.002 for N2 vs. N0) (all *p <*0.05).

**Table 1:** Association of HMGB3 expression with clinicopathologic characteristics by logistic regression.

Clinical characteristics	Total(n)	Odds ratio for HMGB3 expression	P value
Age (>60 vs. ≤60)	1082	1.074 (0.845-1.365)	0.559
ER status (Positive vs. Negative)	1033	0.207 (0.147-0.287)	<0.001
PR status (Positive vs. Negative)	1030	0.393 (0.300-0.514)	<0.001
HER2 status (Positive vs. Negative)	715	1.550 (1.085-2.226)	0.017
T classification			
(T2 vs. T1)	906	1.126 (0.849-1.495)	0.409
(T3 vs. T1)	416	0.787 (0.521-1.185)	0.253
(T4 vs. T1)	312	2.328 (1.122-5.118)	0.028
N classification			
(N1 vs. N0)	872	0.999 (0.762-1.308)	0.992
(N2 vs. N0)	630	1.793 (1.189-2.730)	0.006
(N3 vs. N0)	590	1.002 (0.617-1.623)	0.994
M classification			
(M1 vs. M0)	922	1.784 (0.724-4.787)	0.221
Clinical stage			
(Stage II vs. I)	800	1.114 (0.800-1.555)	0.522
(Stage III vs. I)	423	1.297 (0.882-1.910)	0.187
(Stage IV vs. I)	199	1.815 (0.683-5.129)	0.239

### Analysis of the relationship between HMGB3 expression and survival outcomes

**Figure 4:**
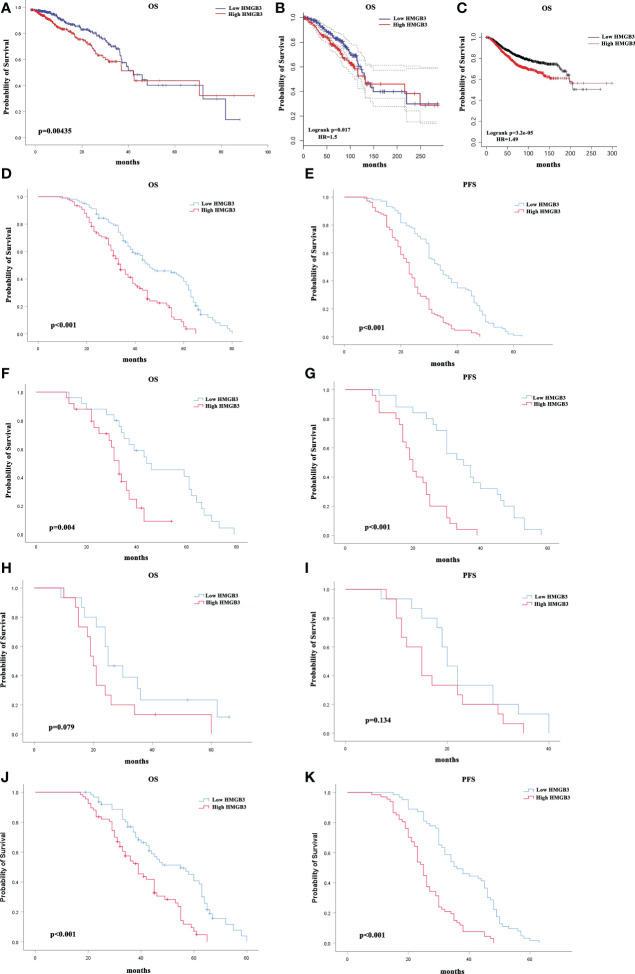
Correlation between breast cancer survival prognosis and HMGB3 gene expression. **(A)** With the OncoLnc web tool, the relationship between HMGB3 expression and breast cancer patient prognosis was analyzed. **(B)** The GEPIA database was used to analyze survival data. **(C)** HMGB3 survival analysis using Kaplan-Meier Plotter software. Survival analysis of a separate cohort of breast cancer patients treated in our center: Kaplan-Meier survival curves for OS **(D)** and Kaplan-Meier survival curves for PFS in breast cancer patients **(E)**. Kaplan-Meier survival curves for OS in subgroups of this cohort stratified by HER2 positivity **(F)**, TNBC **(H)**, and ER positivity **(J)** in breast cancer patients. Kaplan-Meier survival curves for PFS in subgroups stratified by HER2 positivity **(G)**, TNBC **(I)**, and ER positivity **(K)** in breast cancer patients.

To determine whether the survival of BRCA patients was correlated with HMGB3 expression, we divided the patients into high (50%) and low (50%) HMGB3 expression groups and further compared the OS of the two groups using the OncoLnc online tool. After controlling for other important variables, the findings of regression and multivariate analyses revealed an association with survival. Notably, HMGB3 expression had a remarkable impact on the prognosis of BRCA. In addition, there was a statistically significant correlation between HMGB3 and prognosis and overexpression of HMGB3 was linked to a worse OS. Poor prognosis in BRCA was linked to high HMGB3 expression (p=0.00435) (**Figure 4A**).

We also analyzed the correlation between HMGB3 expression levels and cancer prognosis in the GEPIA database. In the majority of cases, the hazard ratio (HR) was used in survival analysis. High expression of HMGB3 was related to a poor outcome in BRCA, according to the survival curves (OS: HR=1.5, p=0.017) (**Figure 4B**). The results above were also confirmed using Kaplan-Meier Plotter database analysis (OS (n=1879): HR=1.49, p<0.001) (**Figure 4C**). In addition, we evaluated the value of HMGB3 in predicting the progression-free survival (PFS) and OS of breast cancer patients in our center. Kaplan-Meier survival analysis demonstrated that patients with low HMGB3 expression had better survival outcomes in terms of PFS (p=0.022) and OS (p<0.001) (**Figures 4D, E**). A total of 210 patients were further divided into three types: TNBC, HER2 positive, and ER positive. We further analyzed whether the expression of HMGB3 was related to the prognosis of these different types. The results showed that high HMGB3 expression predicted worse OS outcomes in patients with HER2 positivity (p=0.004) (**Figure 4F**) and ER positivity (p<0.001) (**Figure 4J**). In contrast, there was no difference in the OS survival outcome between TNBC patients with low and high HMGB3 expression (p=0.079) (**Figure 4H**). However, the analysis of PFS revealed that high expression of HMGB3 was associated with poor survival in patients with the HER2-positive and ER-positive breast cancer subtypes (p<0.001) (**Figures 4G, K**), but not in those with the TNBC breast cancer subtype (p=0.134) (**Figure 4I**). These findings revealed that high HMGB3 expression could potentially be used as a prognostic biomarker in BRCA.

### Multivariate and univariate cox regression analysis

**Table 2** presents the findings of univariate and multivariate Cox analyses of survival rates in patients with BRCA in the TCGA database. T stage (p=0.012), N stage (p<0.001), M stage (p<0.001), age (p<0.001), pathologic stage (p<0.001) and HMGB3 expression (p=0.044) were linked with OS in patients with BRCA in univariate Cox analysis of HMGB3. In multivariate Cox regression analysis of the aforementioned factors, N stage (p=0.015), M stage (p=0.041), age (p<0.001), pathological stage (p=0.033), and high HMGB3 expression (p=0.043) were discovered to be independent risk variables affecting survival in BRCA patients.

**Table 2:** Univariate and multivariate Cox analyses of overall survival in breast cancer.

Characteristics	Univariate analysis		Multivariate analysis	
	HR (95% CI)	*P* value	HR (95% CI)	*P* value
T stage (T4 and T3 vs. T2 and T1) N stage (N3, N2 and N1vs N0)	0.622 (0.429-0.901)	0.012	0.476 (0.248-0.915)	0.699
	0.447 (0.313-0.638)	<0.001	0.740 (0.398-1.377)	0.015
M stage (M1 vs. M0)	0.235 (0.136-0.405)	<0.001	0.332 (0.129-0.852)	0.041
PR status (Positive vs. Negative)	1.367 (0.977-1.912)	0.068	1.131 (0.523-2.446)	0.754
ER status (Positive vs. Negative)	1.405 (0.977-2.019)	0.066	1.883 (0.823-4.309)	0.134
HER2 status (Positive vs. Negative)	0.628 (0.383-1.028)	0.064	0.993 (0.558-1.765)	0.980
Age (≤60 vs. >60)	2.020 (1.465-2.784)	<0.001	3.272 (1.982-5.401)	<0.001
Pathologic stage (Stage IV and III vs. Stage I and II)	0.418 (0.298-0.587)	<0.001	0.471 (0.225-0.986)	0.033
HMGB3 (High vs. Low)	0.720 (0.523-0.991)	0.044	0.773 (0.460-1.297)	0.043

To further determine whether the expression and clinical characteristics of HMGB3 can be regarded as independent risk factors for BRCA, we conducted univariate and multivariate Cox regression analyses in our center patients. The univariate analysis indicated that T stage (p<0.001), N stage (p<0.001), ER status (p=0.008), age (p<0.001) and HMGB3 expression (p<0.001) were significantly associated with decreased OS (**Table 3**). Multivariate Cox analysis revealed that T stage (p<0.001), ER status (p=0.02), age (p<0.001) and HMGB3 expression (p<0.001) were independent predictors of OS. Univariate analysis indicated that T stage (p<0.001), N stage (p<0.001), ER status (p=0.002), PR status (p=0.029), age (p<0.001) and HMGB3 expression (p<0.001) were significantly associated with decreased PFS (**Table 4**). Multivariate Cox analysis revealed that T stage (p<0.001), N stage (p=0.015), age (p<0.001) and HMGB3 expression (p<0.001) were independent predictors of PFS.

**Table 3:** Cox regression analysis of overall survival in patients with metastatic breast cancer.

Characteristics	Univariate analysis		Multivariate analysis	
	HR (95% CI)	*P* value	HR (95% CI)	*P* value
T stage (T4 and T3 vs. T2 and T1)	4.943 (3.817-6.401)	<0.001	4.656 (3.551-6.106)	<0.001
N stage (N3, N2 and N1 vs. N0)	1.655 (1.258-2.176)	<0.001	1.328 (0.980-1.800)	0.067
HER2 status (Positive vs. Negative)	1.055 (0.741-1.503)	0.766		
ER status (Positive vs. Negative)	0.657 (0.482-0.896)	0.008	0.681 (0.493-0.941)	0.02
PR status (Positive vs. Negative)	0.838 (0.617-1.138)	0.258		
Age (≤50 vs.>50)	0.481 (0.350-0.661)	<0.001	0.538 (0.379-0.762)	<0.001
HMGB3 (High vs. Low)	2.308 (1.651-3.226)	<0.001	2.603 (1.744-3.886)	<0.001

**Table 4:** Cox regression analysis of progression-free survival in patients with metastatic breast cancer.

Characteristics	Univariate analysis		Multivariate analysis	
	HR (95% CI)	*P* value	HR (95% CI)	*P* value
T stage (T4 and T3 vs. T2 and T1)	3.383 (2.727-4.198)	<0.001	3.223 (2.565-4.050)	**<0.001**
N stage (N3, N2 and N1 vs. N0)	1.818 (1.404-2.354)	<0.001	1.414 (1.069-1.869)	**0.015**
HER2 status (Positive vs. Negative)	1.090 (0.792-1.498)	0.597		
ER status (Positive vs. Negative)	0.647 (0.488-0.857)	0.002	0.846 (0.483-1.481)	0.557
PR status (Positive vs. Negative)	0.738 (0.561-0.970)	0.029	0.774 (0.451-1.329)	0.352
Age (≤50 vs.>50)	0.547 (0.414-0.723)	<0.001	0.558 (0.410-0.748)	<0.001
HMGB3 (High vs. Low)	2.840 (2.100-3.842)	<0.001	3.309 (2.321-4.718)	<0.001

### Relationship between tumor-infiltrating immune cells and HMGB3 expression

**Figure 5:**
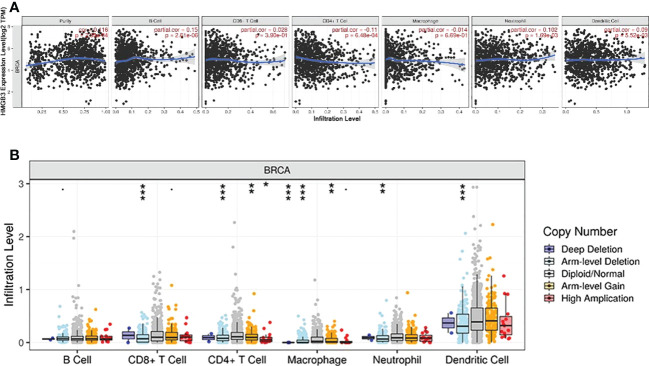
Correlation between breast cancer immune cell infiltration levels and HMGB3 expression. **(A)** Relationship between high levels of immunological infiltrates and HMGB3 expression. **(B)** Relationship between HMGB3 somatic copy number variations and the volume of immunological infiltrates. *p<0.05, **p<0.01, ***p<0.001.

Previous research has demonstrated that the amount and activity of lymphocytes that infiltrate tumors affect the survival of some cancer patients (23, 24). Therefore, we sought to ascertain whether HMGB3 expression is associated with immune cell infiltration in BRCA using the TIMER2.0 database. The examination of immune infiltration in clinical cancer cases is significantly influenced by the tumor purity. **Figure 5A** shows a positive relationship between HMGB3 expression and tumor purity in BRCA (cor=0.116, p=2.05e-04). Additionally, we discovered a positive relationship between the expression of HMGB3 and B cells (cor=0.15, p=2.61e-06), dendritic cells (cor=0.09, p=5.52e-03), and neutrophils (cor=0.102, p=1.69e-03), but a negative relationship with CD4+ T cells (cor=-0.11, p=6.48e-04). CD8+ T cells or macrophages were not significantly correlated with HMGB3 expression (p>0.05). Furthermore, five invading immune cells were substantially associated with the arm-level ablation of HMGB3. However, the prevalence of immunological infiltrates in B cells was unrelated to somatic copy number changes (**Figure 5B**). These findings suggest that HMGB3 is essential for BRCA immune infiltration.

### Analysis of HMGB3 gene alterations and gene coexpression in BRCA

**Figure 6:**
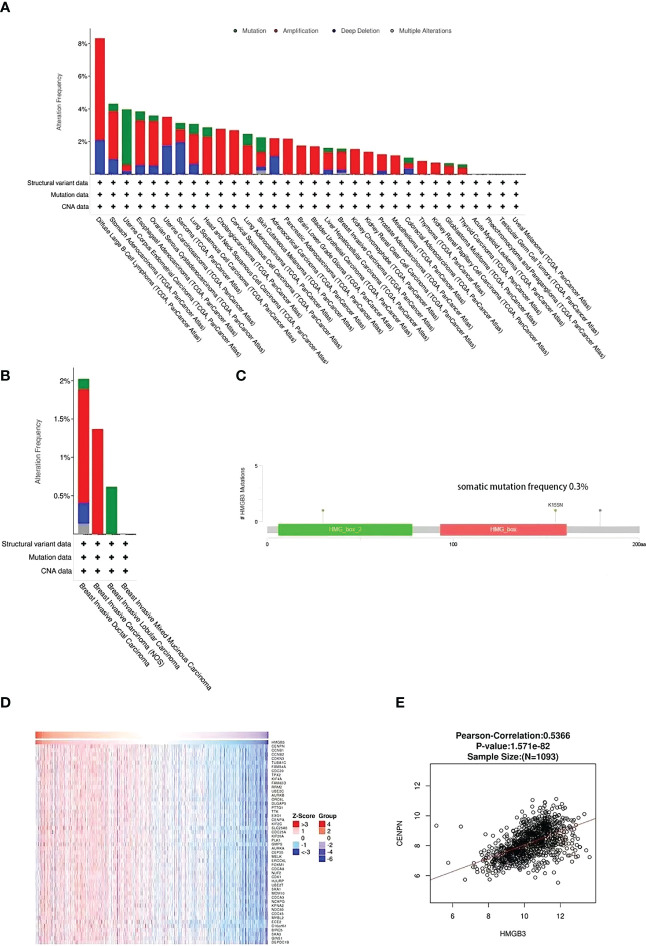
HMGB3 coexpression and mutational characteristics in breast cancer. **(A)** Pan-cancer frequency variations due to HMGB3 mutation type. **(B)** The frequency and kind of HMGB3 genetic alterations found in various breast cancer types. **(C)** Analysis of breast cancer HMGB3 mutations. **(D)** The heap maps highlight the genes that have a positive correlation to HMGB3 in breast cancer. **(E)** LinkedOmics database analysis of the relationship between HMGB3 and CENPN expression in breast cancer.

The genetic variation of HMGB3 in different types of malignancies was analyzed using cBioPortal. Approximately 1% of 10967 samples had HMGB3 gene changes. Top two types of cancer for HMGB3 modification frequency were diffuse large B-cell lymphoma (8.33%) and stomach adenocarcinoma (4.32%), as illustrated in **Figure 6A**. Genetic alterations of HMGB3 in BRCA samples consisted of mutations, deep deletions, amplifications, and multiple alterations. Among the different forms of breast cancer, invasive ductal carcinoma of the breast showed the greatest frequency of change (2.02%), followed by invasive carcinoma of the breast and invasive lobular carcinoma of the breast (**Figure 6B**). The frequency of somatic HMGB3 mutations in breast cancer samples was 0.3%. The two mutations were discovered to be missense mutations with K155N and K30N protein alterations (**Figure 6C**). Additionally, we investigated the coexpression of HMGB3 using the LinkedOmics repository. The top 50 important positively coexpressed genes are shown in the heatmap in **Figure 6D**. The most significant positive gene was CENPN (cor=0.5366, p=1.571e-82) (**Figure 6E**).

### Enrichment analyses of genes coexpressed with HMGB3

**Figure 7:**
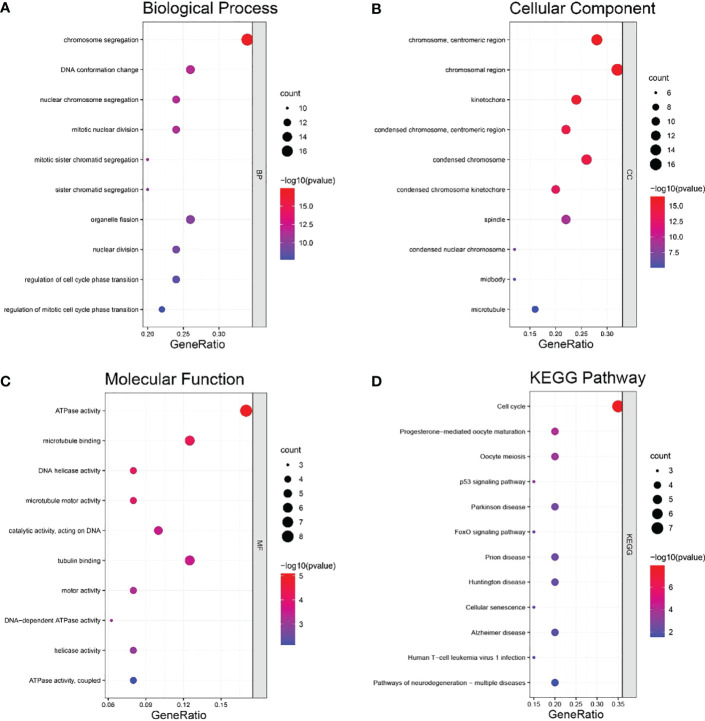
Coexpressed genes for HMGB3 were analyzed for GO and KEGG enrichment analyses. **(A)** Analysis of positively connected genes’ GO BP enrichment. **(B)** Analysis of positively associated genes’ GO CC enrichment. **(C)** Analysis of positively linked genes’ GO MF enrichment. **(D)** Enrichment analysis of KEGG pathways for the positively related genes.

DAVID was used to conduct GO and KEGG enrichment analyses. The HMGB3 coexpressed genes were primarily enriched in chromosome segregation, DNA conformation change, and nuclear chromosome segregation, according to the GO biological process (BP) analysis (**Figure 7A**). In the GO cellular component (CC) analysis, the HMGB3 coexpressed genes were highly correlated with chromosome centromeric region, chromosomal region, and kinetochore (**Figure 7B**). The enrichment analysis of GO molecular functions revealed a substantial correlation between the coexpressed genes and ATPase activity, microtubule binding, and DNA helicase (MF) (**Figure 7C**). A substantial link between the coexpressed genes and the cell cycle, progesterone-mediated oocyte maturation, oocyte meiosis, and p53 signaling pathways was found by KEGG pathway enrichment analysis (**Figure 7D**).

## Discussion

Women are more likely to die of breast cancer than any other cancer in the world. Searching for critical biomarkers for the diagnosis and treatment of breast cancer has emerged as a popular research area with the rapid development of genomics and molecular biology. Additionally, recent studies have demonstrated that clinicopathological characteristics such as age and stage are insufficient to reliably predict a cancer patient’s prognosis. Therefore, more research is still needed to fully understand the molecular pathways underlying tumor development and prognosis. High mobility group (HMG) proteins are estimated to be the second most prevalent proteins in cells and are crucial for chromatin domain formation. HMG proteins interact with nucleosomes, nucleosome complexes, transcription factors, and histone H1 to facilitate transcriptional fine-tuning in response to fast environmental changes (25). HMGB3 is a member of the HMG family. Studies have revealed that HMGB3 can accelerate the growth of a number of tumors, including endometrial cancer, gastric carcinoma, and cervical cancer, and is a standalone indicator of poor prognosis (26–28). At the same time, Xie et al. suggested that HMGB3 promotes bladder tumor cell proliferation and invasion by downregulating microRNA-532-5p through the HMGB3/Wnt/β-catenin signaling pathway (29).

The prognostic role of HMGB3 in BRCA has not been demonstrated, although it has been proven to have a role in the growth of a number of tumors, and its prognostic role has been highlighted. Li et al. discussed the significant increase in HMGB3 in tamoxifen-resistant breast cancer (30). Several experiments showed that the upregulation of HMGB3 leads to the malignant behavior of BRCA (10, 31). Other research demonstrated that HDAC3 enhances immune escape in BRCA by suppressing microRNA-130a-3p and upregulating HMGB3 expression (32). In our research, we examined the possible mechanisms through which HMGB3 promotes BRCA and the potential utility of HMGB3 as a predictive biomarker. A pan-cancer investigation revealed elevated HMGB3 expression in a number of malignancies, including breast carcinoma. For additional verification, we used the GEPIA and UALCAN databases. According to the bc-GenExMiner web tool, HMGB3 expression was strongly linked with HER2 status, the triple-negative subtype, the basal-like subtype, NPI, SBR grade, and nodal status. In contrast to normal tissue samples, BRCA samples showed an inverse correlation between HMGB3 levels and PR and ER statuses. We also used logistic regression to analyze the relationship between HMGB3 expression levels and the clinicopathological features of BRCA. The findings revealed a substantial relationship between HMGB3 expression levels and ER, PR, HER2, T and N classifications. As a result, these findings revealed that HMGB3 expression may be able to predict the outcome of BRCA.

Previous research demonstrated that HMGB3 overexpression is connected to a poor prognosis in bladder cancer and esophageal cancer (6, 9). Therefore, we further examined the connection between HMGB3 expression and BRCA prognosis using specimens from the OncoLnc, GEPIA and Kaplan-Meier Plotter databases to determine if HMGB3 expression is connected to the prognosis of BRCA. The findings revealed that increased expression of HMGB3 was related to a poor prognosis and that HMGB3 can be used as a predictive biomarker in BRCA.

Immune cell infiltration has been shown to be a predictive indicator of cancer progression in previous studies (33, 34). Additionally, although it has not been confirmed, several studies showed that the expression of HMGB3 may be a promising immunological target. Therefore, we assumed that HMGB3 expression and immunological infiltration were connected. A study on immune infiltration in BRCA revealed a substantial association between HMGB3 and biomarkers for immune infiltrating cells. We discovered a positive connection between HMGB3 expression and B cells, dendritic cells, and neutrophils. Studies suggested that B cells and dendritic cells in the tumor microenvironment can activate antitumor responses (35, 36). P53, the first identified tumor suppressor gene (37), plays a crucial role in controlling the cell cycle and inhibiting angiogenesis and apoptosis. Notably, KEGG enrichment analysis revealed enrichment in the cell cycle, oocyte meiosis, progesterone-mediated oocyte maturation, and p53 signaling pathways. Through univariate and multivariate Cox regression analyses, we also examined the effect of risk variables on BRCA. The findings demonstrated that elevated HMGB3 expression was a distinct predictive factor for BRCA.

In summary, HMGB3 might be an independent biomarker of poor prognosis in BRCA patients. Our research is based on the findings of numerous web databases and its results were also validated in clinical samples and *in vitro* experiments, but more clinical case data are still needed for further verification. More research on the clinical function of HMGB3 in BRCA and its immune escape mechanism is still needed. The results of this research may provide potential reference value for future studies on the function of HMGB3 in BRCA.

## Data availability statement

The datasets presented in this study can be found in online repositories. The names of the repository/repositories and accession number(s) can be found in the article/Supplementary Material.

## Ethics statement

This research was checked and approved by the Ethics Committee of Hubei Cancer Hospital (approval number LLHBCH2020LW-022).

## Author contributions

BL and XZ designed the study. XZ performed the bioinformatics analysis and wrote the manuscript. QZ, GL, and XL offered critical feedback. The final manuscript was read and agreed upon by all authors.
