# Supplementary material for: Overexpression of HMGB3 and its prognostic value in breast cancer
Source: Front Oncol. 2022 Dec 22;12:1048921. doi: 10.3389/fonc.2022.1048921 (PMC9815698; doi:10.3389/fonc.2022.1048921)
Supplement: Supplementary file 1 [file Table_1.docx]

| Table S1: Clinical data of 210 case of metastatic breast cancer patient | | | | | | |
| --- | --- | --- | --- | --- | --- | --- |
| Characteristics | |  |  |  |  | N（%） |
| Age |  |  |  |  |  |  |
| ≤50 |  |  |  |  |  | 95(45%) |
| ＞50 |  |  |  |  |  | 115(55%) |
| ER status |  |  |  |  |  |  |
| Negative |  |  |  |  |  | 80(38%) |
| Positive |  |  |  |  |  | 129(62%) |
| PR status |  |  |  |  |  |  |
| Negative |  |  |  |  |  | 96(46%) |
| Positive |  |  |  |  |  | 114(54%) |
| HER2 status | |  |  |  |  |  |
| Negative |  |  |  |  |  | 160(76%) |
| Positive |  |  |  |  |  | 50(24%) |
| T classification | |  |  |  |  |  |
| T1/T2 |  |  |  |  |  | 110(52%) |
| T3/T4 |  |  |  |  |  | 100(48%) |
| N classification | |  |  |  |  |  |
| N0/N1 |  |  |  |  |  | 93(44%) |
| N2/N3 |  |  |  |  |  | 117(56%) |

Table S2: Correlation between HMGB3 expression and clinicopathologic parameters of breast cancer

| Characteristics |  | HMGB3 expression | |  | *P* value |
| --- | --- | --- | --- | --- | --- |
|  |  | Low(n=103) | High(n=107) |  |  |
| Age |  |  |  |  |  |
| ≤50 |  | 48 | 47 |  | 0.401 |
| ＞50 |  | 55 | 60 |  |  |
| ER status |  |  |  |  |  |
| Positive |  | 63 | 66 |  | 0.47 |
| Negative |  | 40 | 41 |  |  |
| PR status |  |  |  |  |  |
| Positive |  | 54 | 60 |  | 0.348 |
| Negative |  | 49 | 47 |  |  |
| HER2 status |  |  |  |  |  |
| Positive |  | 25 | 25 |  | 0.503 |
| Negative |  | 78 | 82 |  |  |
| T classification |  |  |  |  |  |
| T1/T2 |  | 63 | 57 |  | 0.155 |
| T3/T4 |  | 40 | 50 |  |  |
| N classification |  |  |  |  |  |
| N0/N1 |  | 46 | 47 |  | 0.513 |
| N2/N3 |  | 57 | 60 |  |  |
